# Supplementary material for: Optimization of Ultrasonic-Assisted Extraction Conditions for Bioactive Components and Antioxidant Activity of Poria cocos (Schw.) Wolf by an RSM-ANN-GA Hybrid Approach
Source: Foods. 2023 Feb 1;12(3):619. doi: 10.3390/foods12030619 (PMC9914185; doi:10.3390/foods12030619)
Supplement: Supplementary file 1 [file foods-12-00619-s001.zip › foods-2108876-supplementary.pdf]

Supplementary data

Optimization of ultrasonic-assisted extraction conditions for bio-active components and antioxidant activity of Poria cocos (Schw.) Wolf by RSM-ANN-GA hybrid approach

Table S1. The multiple response monitoring parameters of four triterpenes acids

Compound	Parention（m/z）	Daughterion (m/z)	DP（eV）	EP（eV）	CE（eV）	CXP（eV）
Pachymic acid	527.20	405.20	200	10	55	11
		465.20*	200	10	55	11
Dehydrotrametenolic acid	453.20	323.30	200	10	55	11
		337.20*	200	10	55	11
Trametenolic acid	455.30	339.20	200	10	55	11
		425.30*	200	10	55	11
Tsugaric acid A	513.20	391.20	200	10	58	11
		451.50*	200	10	58	11

* Quantitative daughterion ; DP: Declustering Potential; EP: Entrance potential; CE: Collision Energy; CXP: Cell exit potential.

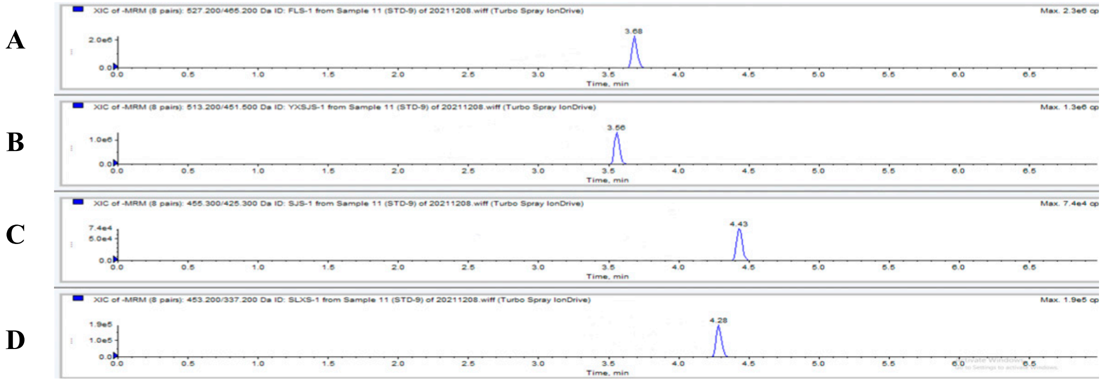

Figure S1. Extracted ion chromatogram (XIC) of MRM for four triterpenes acids (A: pachymic acid; B: tsugaric acid A; C: trametenolic acid; D: dehydrotrametenolic acid;)

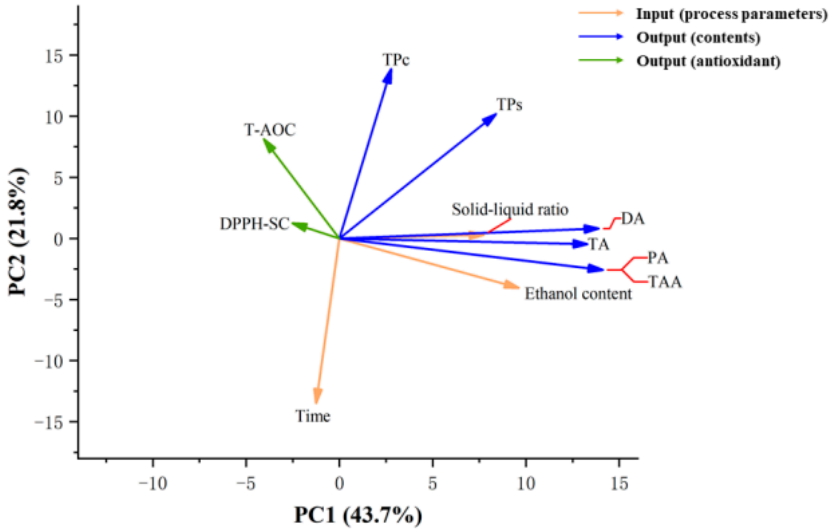

Figure S2. PCA of content-antioxidant profile and optimal topology of a developed ANN model

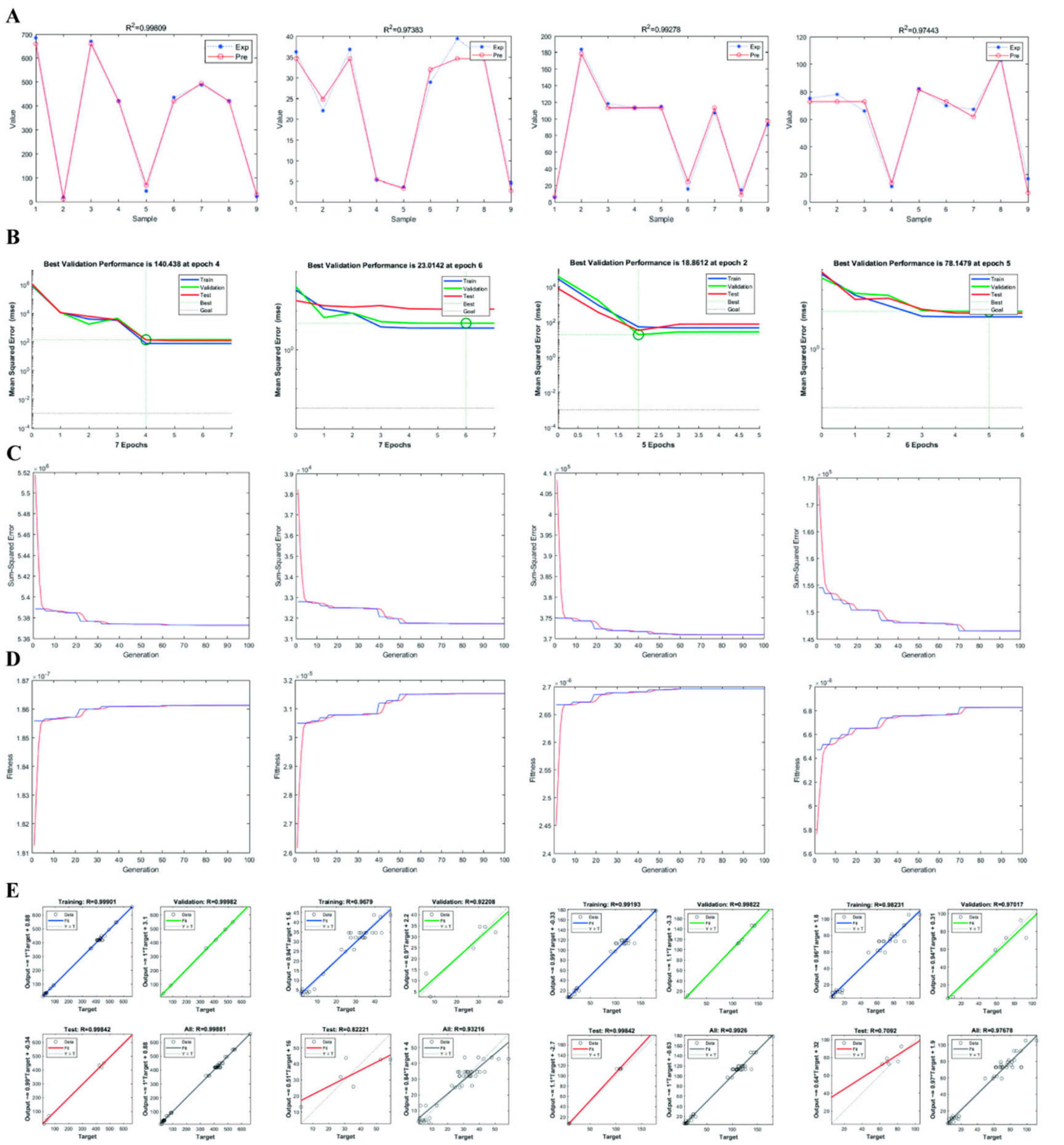

Figure S3. ANN modeling and training. (A,B) ANN training performance of each output in Group 1, from left to right: pachymic acid, trametenolic acid, tsugaric acid A and dehydrotrametenolic acid (same as below), (C,D) GA algorithm optimization ANN model, (E) Regression of experimental and predicted values in ANN model.

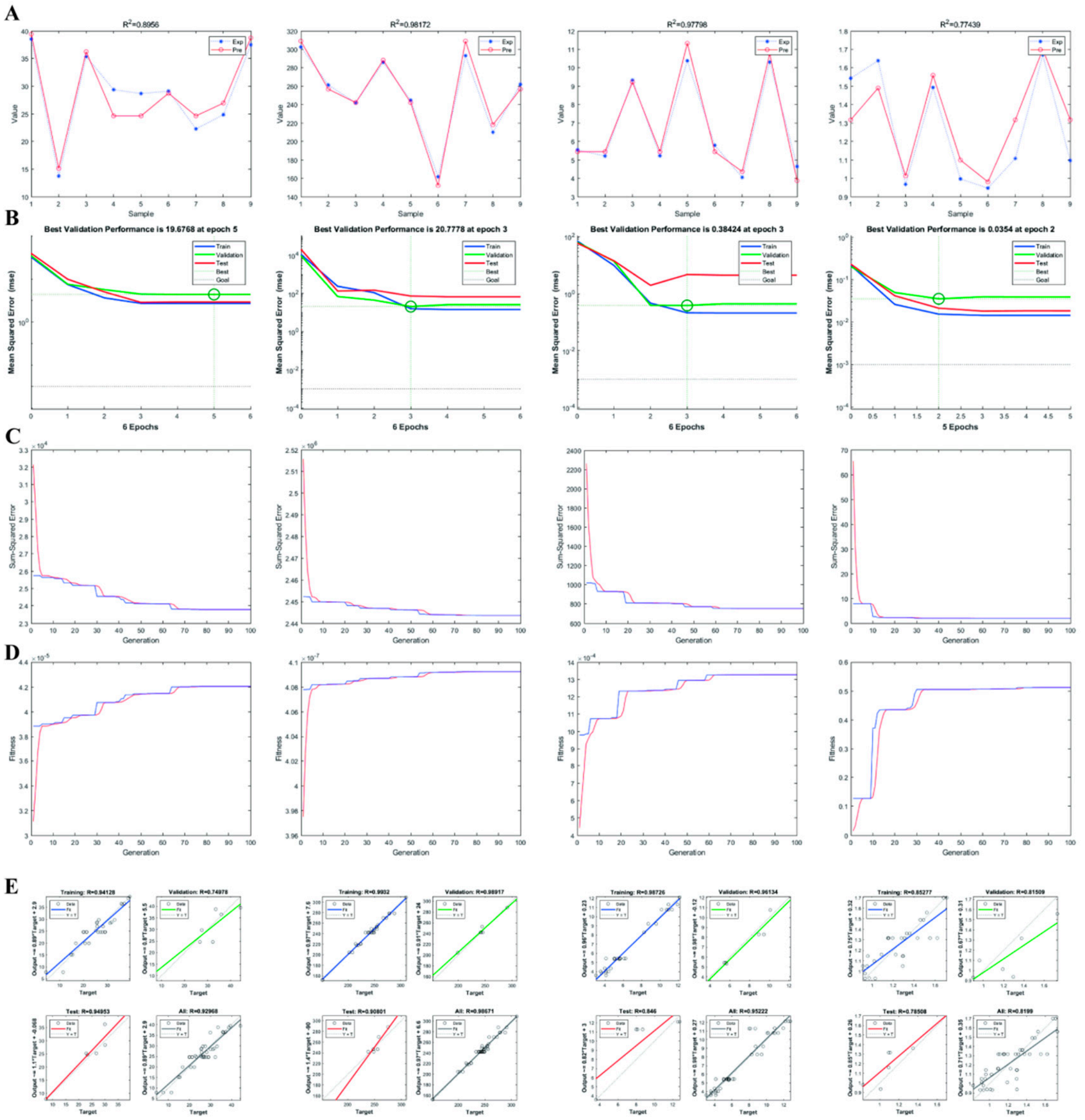

Figure S4. ANN modeling and training. (A,B) ANN training performance of each output in Group 2, from left to right: total polysaccharide content, total phenolic content, DPPH radical scavenging and total antioxidant capacity (same as below), (C,D) GA algorithm optimization ANN model, (E) Regression of experimental and predicted values in ANN model.
